# Development of New Potential Inhibitors of β1 Integrins through In Silico Methods—Screening and Computational Validation

**DOI:** 10.3390/life12070932

**Published:** 2022-06-22

**Authors:** Disraeli Vasconcelos, Beatriz Chaves, Aline Albuquerque, Luca Andrade, Andrielly Henriques, Geraldo Sartori, Wilson Savino, Ernesto Caffarena, João Herminio Martins-Da-Silva

**Affiliations:** 1 Laboratório de Biologia Estrutural e Funcional em Biofármacos, Fundação Oswaldo Cruz Ceara, Eusebio 61773-270, Brazil; disraeli.vasconcelos@fiocruz.br (D.V.); beatriz.chaves@ioc.fiocruz.br (B.C.); aline.oalbuquerque@fiocruz.br (A.A.); lucaandrade@aluno.fiocruz.br (L.A.); andriellycosta@aluno.fiocruz.br (A.H.); geraldo.sartori@fiocruz.br (G.S.); 2 Laboratório de Pesquisa sobre o Timo, Instituto Oswaldo Cruz, Fundação Oswaldo Cruz, Rio de Janeiro 21040-360, Brazil; wilson.savino@fiocruz.br; 3 Instituto Nacional de Ciência e Tecnologia em Neuroimunomodulação, Instituto Oswaldo Cruz, Fundação Oswaldo Cruz, Rio de Janeiro 21040-360, Brazil; 4 Rede de Pesquisa em Neuroimunomodulação, Instituto Oswaldo Cruz, Fundação Oswaldo Cruz, Rio de Janeiro 21040-360, Brazil; 5 Grupo de Biofísica Computacional e Modelagem Molecular, Programa de Computação Científica (PROCC), Fundação Oswaldo Cruz, Rio de Janeiro 21040-222, Brazil; ernesto.caffarena@fiocruz.br

**Keywords:** integrin, VLA-4, peptidomimetic inhibitors, molecular docking, de novo design, molecular dynamics, lead optimization, ADMET, MM/PBSA

## Abstract

Integrins are transmembrane receptors that play a critical role in many biological processes which can be therapeutically modulated using integrin blockers, such as peptidomimetic ligands. This work aimed to develop new potential β1 integrin antagonists using modeled receptors based on the aligned crystallographic structures and docked with three lead compounds (BIO1211, BIO5192, and TCS2314), widely known as α4β1 antagonists. Lead-compound complex optimization was performed by keeping intact the carboxylate moiety of the ligand, adding substituents in two other regions of the molecule to increase the affinity with the target. Additionally, pharmacokinetic predictions were performed for the ten best ligands generated, with the lowest docking interaction energy obtained for α4β1 and BIO5192. Results revealed an essential salt bridge between the BIO5192 carboxylate group and the Mg^2+^ MIDAS ion of the integrin. We then generated more than 200 new BIO5192 derivatives, some with a greater predicted affinity to α4β1. Furthermore, the significance of retaining the pyrrolidine core of the ligand and increasing the therapeutic potential of the new compounds is emphasized. Finally, one novel molecule (1592) was identified as a potential drug candidate, with appropriate pharmacokinetic profiles, similar dynamic behavior at the integrin interaction site compared with BIO5192, and a higher predicted affinity to VLA-4.

## 1. Introduction

Cell migration is fundamental for many of the lymphohematopoietic system’s functions, including physiological leukocyte traffic and the genesis and maintenance of different inflammatory responses. Among the molecular interactions that govern leukocyte migration, there are those mediated by selectins, chemokines, and extracellular matrix (ECM) components. The primary cell surface receptors involved in adhesion to the ECM elements belong to the integrin family.

Integrins of the β1 family are expressed by some blood cell types, including T lymphocytes, and play a critical role in a wide range of biological processes and chronic inflammatory diseases [1,2], carcinogenesis, and metastasis [2]. Herein, our interest was focused on integrins α4β1, α5β1, and αVβ1; all of them share the same ECM component, namely fibronectin.

Integrins are noncovalently linked heterodimeric molecules consisting of two subunits called α and β. A total of 18 α and eight β subunits have been found in vertebrates, making at least 24 heterodimers [3]. Therefore, integrins are considered the most diverse family of cell adhesion molecules [4,5]. Located in the α subunit, the β-propeller [6,7] is a typical structural domain that comprises the N-terminal globular region, essential for recognizing specific ligands in integrins that do not possess the so-called I domain [5].

Another relevant structural feature is a region located in the β subunit called the metal ion-dependent adhesion site (MIDAS), which contains up to three divalent cations. According to Nagae and co-workers [7], coordination of the MIDAS ion (Mg^2+^) by a carboxylate group (COO^−^) in the substrate or a ligand is the central mechanism for the interaction between the integrin and specific molecules.

Among the therapeutic approaches involving blocking integrin functions, one of them concerns the use of peptidomimetic ligands, based on most integrins’ capability to recognize peptide moieties to their binding molecules. It was shown that adding a chemical structure to the N-terminal portion of LDV tripeptide would considerably increase its inhibitory capacity. This structure, named 4-[N′-(2-methyl phenyl) ureido] phenyl acetyl (PUPA), brought a new range of compounds, becoming a template for drug development by pharmaceutical companies [8].

Monoclonal antibodies have also been used to treat diseases associated with an abnormal integrin expression, such as vedolizumab [9] and natalizumab [10]. However, the production of these macromolecules demands high associated costs, making the choice of small ligands (lower molecular weight) viable.

Frequently, theoretical and computational knowledge about receptor–ligand recognition adds valuable understanding of the molecular bases governing recognition to plan new drugs with functional therapeutic potential. For example, innovative methods to build novel ligands through smaller molecular fragments (fragment-based de novo design), combined with molecular dynamics (MD) and molecular docking methodologies, configure an initial functional approach for planning and idealizing new prototype compounds [11].

Fragment-based drug discovery (FBDD) has become a practical procedure for exploring the chemical space of a target protein, leading to higher affinity lead compounds [12]. Particularly, fragment-based de novo design involves the creation of new chemical entities based on the structure of the binding site with which they must interact [13]. FBDD projects have some attractive advantages, such as lower investments in research and development and the use of smaller libraries of low-complexity molecules. These advantages have encouraged researchers to develop new inhibitors for diverse protein targets. Since integrins are multidomain proteins, the use of FBDD may be helpful in the search for new binding sites and modulating molecules [14].

Traditionally, drug development occurs by testing newly synthesized compounds in biological assays. However, some adverse factors are commonly found at this stage. Several analyses have shown that half of the failures were attributed to two factors: (i) inadequate pharmacokinetics (39%) and (ii) toxicity in animals (11%) [15]. Hence, there is a growing demand for data on compounds’ absorption, distribution, metabolism, elimination, and toxicity (ADMET). Furthermore, it is desired that they have a suitable oral availability in drug candidates’ selection process. To this end, Lipinski’s Rule of Five [16,17] is widely required.

Here, we applied computational techniques involving comparative modeling, molecular docking, de novo design, pharmacokinetic studies, and molecular dynamics to develop novel potential β1 integrin antagonist compounds based on existing peptidomimetic molecules. The generic names of these molecules are BIO1211 [8], BIO5192 [17], and TCS2314 [18], being the first and last derived from the PUPA-LDV structure. Currently, numerous β1 integrin antagonists have been reported [19,20] compounds BIO1211, BIO5192, and TCS2314, chosen as the starting point of this study, showed an in vitro inhibitory potential against VLA-4 (α4β1 or very late antigen-4). However, how these molecules interact with particular molecular targets remains primarily unknown. Thus, our goal is to shed light on these challenging questions.

## 2. Material and Methods

### 2.1. Molecular Modeling

Target sequences of the human α4, αV, and β1 domains were obtained from the NCBI protein database, with the respective accession numbers NCBI NP_000876, NP_002201, and NP_391988. The crystal structure of the α5β1 heterodimer [7] (3VI4), aligned to complexes α4β7 [21] (PDB code: 3V4P) and αVβ3 [22] (PDB code: 4O02), was employed to obtain the α4β1 and αVβ1 structures, respectively, using the PyMOL 1.3 software (the PyMOL Molecular Graphics System, Version 1.3 Schrödinger, LCC, New York, NY, USA). Then, these heterodimers were used as templates to build the new models, with a higher degree of structural refinement, using the Modeller 9.15 software [23].

We built global alignments between the targets and template sequences on the Promals server [24] (details in Supplementary Material). For both α4β1 and αVβ1, we generated 100 models and chose the one with the lowest objective function value [25] for the validation process. Therefore, we applied the Procheck [26], Verify3D [27], and ProSA-web [28] servers to estimate the models’ quality and compatibility.

### 2.2. Molecular Docking

We performed the docking assays on the obtained comparative models using the Autodock Vina [29] program implemented in Pyrx 0.9.2 software [30]. Ligands BIO1211, BIO5192, and TCS2314 were built and energy-minimized in Avogadro [31]. The stereochemistry of the molecules was respected, and the protonation states of the ionizable groups were generated at pH 7.4.

We set the box’s geometric center for the docking grid to be the coordinates of ion Mg^2+^ of the MIDAS motif. The box edges were adjusted to 20 Å, 20 Å, and 25 Å for the x, y, and z axes. We defined an exhaustiveness value of 150 for this work.

The best results were sorted out according to the Vina scoring function. In addition, we ranked the results regarding the Euclidean distances between the oxygen atoms of the ligand’s carboxylate group and the Mg^2+^ ion of the integrin MIDAS region, taking a cut-off radius of 3 Å. Finally, we selected a specific binding mode in which the lead compound would remain in a stretched form at the integrin site to optimize its structure.

To validate the docking protocol, we studied the binding modes of 500 VLA-4 ligands and decoys selected in the Chembl database [32] (ID: CHEMBL1907599). The total amount of decoys contained in the database was 28%. We used the same previous docking grid settings. Thereby, we ranked the best-classified molecules based on the Vina score function (Table S1) and assessed the precision of the method in separating active and decoy compounds by plotting a receiver operating characteristics (ROC) curve to measure the area under the curve (AUC) and the enrichment factor (EF).

### 2.3. De Novo Design

The binding modes identified in the molecular docking were taken as references for constructing the novel ligands using the RACHEL module in the Sybyl-X 2.0 (Tripos Inc., St. Louis, MO, USA) software package framework. The RACHEL module (Real-time Automated Combinatorial Heuristic Enhancement of Lead compounds) is a program used to optimize lead compounds that weakly interact with their target in an automated and combinatorial way.

The fragment structures used as building blocks in generating the new molecules were selected from the ZINC15 database [33], and the ZINC subset was the ZINC building blocks (ZBB). The selected fragments were those with a molecular mass ≤ 300 Da and logP ≤ 2. The total number of selected compounds was 15,364,477.

We used the RACHEL module to perform three optimization procedures, preserving the carboxylate atoms without adding chemical groups because this is a crucial component of integrin for detecting particular molecules. Eventually, the results were analyzed using the RACHEL (pKi) scores, and interactions formed between the new compounds and the integrin were examined. Out of the 697 solutions found by RACHEL, ten were chosen for further analyses. The selection criteria for the molecules followed Lipinski’s Rule of Five.

### 2.4. ADMET Predictions

The ten molecules with the highest scores calculated by the RACHEL software were used to verify their structural properties and toxicity. Then, we chose three tools, namely FAF-Drugs3 [34], ClogP/CMR (Sybyl-X module), and Osiris Property Explorer (http://www.openmolecules.org/datawarrior/ accessed on 5 July 2021) [35] to obtain the ADMET estimates.

### 2.5. Molecular Dynamics Simulations and Binding Free Energy Calculations

We selected the lead compound BIO5192 and two of its derivatives to analyze their stability and the conserved interactions with VLA-4 over time by molecular dynamics. We performed the simulations in the Gromacs software package v. 2018.3 [36,37].

The Antechamber program [38] was used to prepare the ligands using the Gaff2 force field [39] and, later, we applied the acpype [40] script to generate the parameters and topologies to the Gromacs package. The protonation state of the VLA-4 protein was adjusted to pH 7.4 using the H++ tool [41].

Simulations were performed in triplicate, with each replica of 500 ns long, totaling 4.5 µs of data. The interaction energies were treated with the Amber all-atom force field ff99sb [42]. The Verlet cut-off scheme treated Coulomb and van der Waals interactions with a distance cut-off of 10 Å. The Particle Mesh Ewald (PME) [43] method was chosen to treat long-range electrostatics. All bonds were constrained using the LINCS algorithm [44].

The systems were placed in a cubic box and solvated with TIP3P water [45]. The addition of three sodium counterions neutralized the complexes. Then, the systems were minimized following the steepest descent algorithm and then went through equilibration in three steps. In the first one, the NVT ensemble was applied for 100 ps until the temperature reached 300 K. Then, we used two simulation steps in the NPT ensemble: one with position restraints for 100 ps, and the last one applying unrestrained simulation for 1 ns. At the end of the simulations, the RMSD data of the heavy atoms of the ligands and the distances of the COO^−^ group in the ligands, and the MIDAS ion of the integrin were submitted to least-squares fit interpolation and analyzed with the tools of the Gromacs package. The results were plotted in the software GraphPad Prism 8.0.2 (GraphPad Prism version 8.0.2 for Windows, GraphPad Software, San Diego, California USA, www.graphpad.com). At the end of the simulations, the trajectories of each replica were submitted to binding free energy calculations by MM/PBSA, using the GMXPBSA 2.1 to obtain results from the higher affinity complex. The last 250 ns of the simulations were considered.

## 3. Results and Discussion

### 3.1. Integrin Structural Models

**Figure 1:**
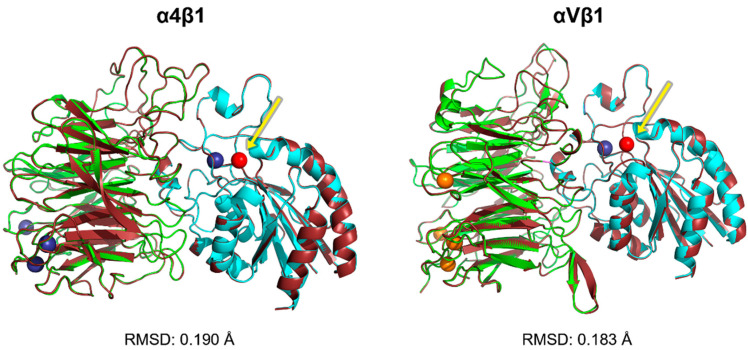
Superimposition between the templates and models generated by Modeller for α4β1 and αVβ1. The template structure is colored in dark red, and the generated models in green (α chain) and cyan (β chain). The dark blue and orange spheres represent the structural divalent calcium and manganese ions. The red sphere, indicated by the arrows, displays the divalent magnesium MIDAS ion present in the integrins β chain for both structures.

In this work, we only modeled the α4β1 and αVβ1 ectodomain interaction site (Figure 1), as we were not interested in the study of allosteric sites or the transmission of signals across the integrin structure. The coverage scores for the α4, αV, and β1 target sequences were 100%, 97%, and 100%, respectively. The percent identity and e-value were 100% and 0, respectively, for all sequences analyzed (Figure S1).

For VLA-4, the RMSD value between template and model structures was 0.190 Å, while for αVβ1, it was 0.183 Å. These subtle differences between both systems were due to more massive refinement of the side chains of amino acids belonging to the interface region of the polypeptide units of the heterodimer. Many of the structural adjustments were managed by Modeller concern loop refinement [46]. (Figure 1). Thus, slight spatial differences between the loops when superimposed are worth noting. Generally, α-helix and β-sheet secondary structures remain aligned, indicating little or no spatial discrepancies.

Regarding the validation by the Ramachandran plot, for α4β1, 88.2% of residues were in favorable regions, plus 11.3% in additional allowed areas, and none in forbidden areas. The same went for αVβ1, where the validated model achieved 88.3% of residues in favorable regions, plus 11.0% in additional allowed areas (Figure S2).

Verify3D indicated that 98.9% of the residues for α4β1 and 98.8% for αVβ1 were compatible with their respective structures. To consider a consistent design, at least 80% of the residues must reach an average score of ≥0.2 (Figure S3). The average scores can be seen in the Supplementary Material.

According to the ProSA-web server, the Z-score plots referring to the validated α4β1 and αVβ1 models denote the quality of the structures. For both proteins, the Z-score was between −5 and −10. In addition, the Z-score values range among the experimentally determined protein chains in the current PDB (Figure S4).

The consensus of the structural validation results estimated by the servers suggests, for both integrins, reliable models are to be used using other computational methodologies, such as molecular docking. As a result, the structures chosen from α4β1 and αVβ1 to be used in the molecular docking process were those created and verified by comparative modeling. Simultaneously, the α5β1 complex was chosen based on the 3VI4 PDB crystal.

### 3.2. Molecular Docking Analysis

**Figure 2:**
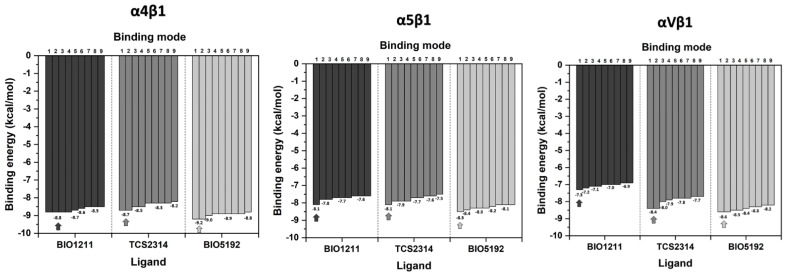
Vina scores for the molecular docking between molecules BIO1211, TCS2314, and BIO5192 and α4β1, α5β1, and αVβ1. The arrows represent the lowest energy values.

Analyses of each of the three ligand binding modes within the active site of the three integrins of the β1 family were carried out (Figure 2). BIO5192 showed the highest affinities (lowest energies) in all studied receptors. The one with the lowest Vina score was for the α4β1 complex, resulting in −9.2 kcal/mol.

On the other hand, BIO1211 exhibited a more significant difference between the values of the Vina score than the other two compounds (BIO5192 and TCS2314) for αVβ1, with lower affinities for the receptor (Figure 2). In addition, all poses showed the ligands bending in the V1 pocket, making it difficult to better fit the site while maintaining the interaction between the COO^−^ group and the Mg^2+^ MIDAS ion (Supplementary Material (Figure S5)).

**Figure 3:**
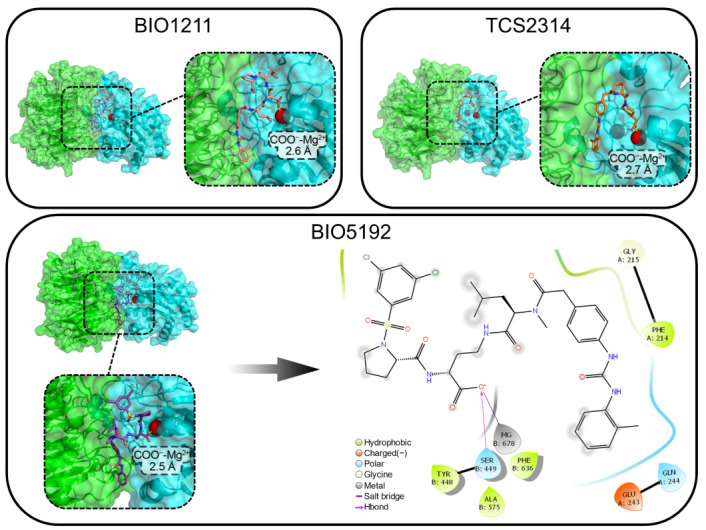
Binding modes from the molecular docking of BIO1211, TCS2314, and BIO5192 in the VLA-4 molecule. The ion of the MIDAS region is represented as a red sphere. The numbers indicate the distance (Angstrom) between the carboxylate oxygen atmosphere and the Mg^2+^ ion of the MIDAS region in the integrin. The image displayed by the black arrow in the lower right corner shows the main interactions between BIO5192 and VLA-4. were produced using the PyMOL and Maestro 11.8, with a 4 Å cut-off radius.

As the compounds’ affinities for the α4β1 heterodimer were remarkable, we analyzed the geometries that presented the closest interaction between the carboxylate oxygens and the Mg^2+^ ion (Figure 3).

Therefore, we noticed that the molecule BIO5192, in addition to having the shortest distance (~2.5 Å) from the carboxylate oxygen to the Mg^2+^ ion, also showed a slightly lower binding score for the α4β1 integrin (−8.9 kcal/mol) compared with the other two compounds, BIO1211 (−8.7 kcal/mol) and TCS2314 (−8.7 kcal/mol). The BIO5192 ligand remained in a stretched conformation when docked to α4β1. According to the literature, this geometry is preferred by peptidomimetic antagonists of VLA-4 integrins [47].

However, the same does not hold for BIO5192 interacting with α5β1 and αVβ1. In this case, the ligand does not fit perfectly into the site, as seen in all the poses in both receptors, hampering the interaction with the targets (Supplementary Material, Figure S6). Thus, these docking analyses ruled out the use of any standard BIO5192 binding mode for α5β1 and αVβ1 for the design of new derivative compounds.

From these analyses, we have chosen the binding mode of the α4β1/BIO5192 complex as the standard model for the next stage of designing new potential integrin inhibitors (Figure 3, right down). It is possible to observe two specific interactions: the carboxylate oxygen of BIO5192 participates in a salt bridge with the MIDAS ion (Mg^2+^) and a hydrogen bond with Ser 449, belonging to the beta chain. Potent VLA-4 antagonists should present a hydrogen bond acceptor, e.g., a carboxylate moiety and hydrophobic groups, to accommodate the pockets formed by nonpolar amino acids [20].

**Figure 4:**
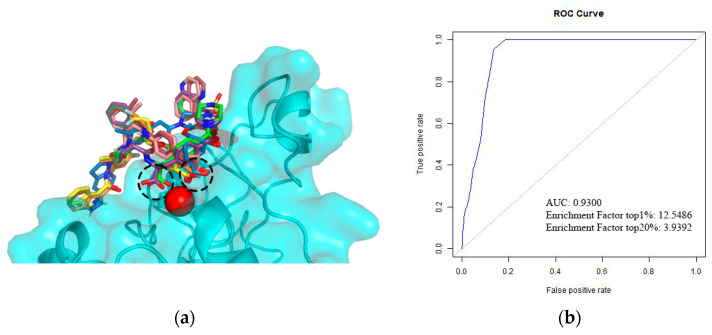
(**a**) Binding modes of the best-ranked VLA-4 ligands selected from the Chembl database. The dashed circles indicate the molecules’ carboxylate group, and the red sphere corresponds to the MIDAS Mg^2+^ ion in the beta chain of the integrin (cyan surface and cartoon). The image was produced using PyMOL; (**b**) ROC curve of the docking protocol and the calculated parameters AUC and enrichment factor for the top 1% and top 20% of the ligands database.

The docking validation results suggest a good performance of the method in separating the active compounds from decoys through the AUC of 0.93 (Figure 4b). Furthermore, the EF value = 12.55 shows the docking’s ability to early rank the actives in the top 1% of the database. Furthermore, the poses of the best-ranked molecules maintained the salt bridge interaction between the carboxylate oxygen and the MIDAS ion Mg^2+,^ and all the ligands remained in a stretched conformation within the binding pocket.

### 3.3. De Novo Design of the Novel BIO5192 Derivatives

**Figure 5:**
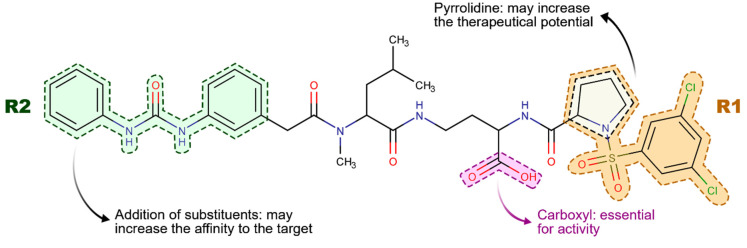
Representation of the BIO5192 ligand moieties subjected to optimization using the RACHEL module.

For the design of the new molecules using the RACHEL module, we kept the BIO5192 carboxylate moiety unaltered. We varied some specific chemical groups, producing three different fragment-based drug design derivatizations (Figure 5). The aim was to create new attractive interactions and ensure higher ligand-receptor affinity.

In the first experiment (I), RACHEL added fragments of the pyrrolidine ring of the ligand BIO5192 replacing the R1 group (Figure 5). In the second derivatization (II), the R2 region was optimized, focusing on the methyl attached to one of the aromatic rings (Figure 5). Finally (III), fragments were added to both moieties simultaneously. RACHEL generated 175, 226, and 296 compounds for I, II, and III derivatization procedures.

According to these results, out of the ten molecules with more significant scores, calculated as pKi, derivatization II produced ligands with higher estimated affinities to the α4β1 molecule (Table 1), and their respective chemical structures can be seen in Figure S7. Notably, a heterocycle moiety in integrin inhibitors, such as a pyrrolidine group, may increase its therapeutic potential [48].

For converting *Ki* values to Gibbs free energy (Δ*Gi*) in kcal/mol, Equation (1) was used:

(1) ∆Gi=RTlnKi

where *R* is the Boltzmann constant and *T* is the absolute temperature; we observe lower energies concerning the docking of BIO5192 in α4β1, which initially achieved −8.9 kcal/mol (binding mode chosen for optimization). Thus, for the ligand 212, the score of 9.08 as pKi, converted through the Gibbs equation with R = 1.99 cal/mol.K and T = 300 K, resulted in ~−13.47 kcal/mol. On the other hand, the tenth ligand with the highest score, code 3604, calculated Δ*Gi* was ~−11.61 kcal/mol.

Therefore, maintaining the proline mimetic moiety in BIO5192 and optimizing other regions resulted in potential potent antagonists with superior affinities to their receptors than the original ligand BIO5192.

### 3.4. Pharmacokinetic Analysis of the Novel BIO5192 Derivatives

The new ten best-ranked derivatives were submitted to ADMET analyses, and Lipinski’s Rule of Five was used to classify ligands.

Considering the results proposed by ClogP/CMR, most ligands presented adequate ClogP measures (ClogP < 5), suggesting the probability of being well absorbed by gastrointestinal barriers (Table S2). High values of this parameter may indicate low absorption and permeability [49].

The FAF-Drugs3 server revealed that compounds have high oral bioavailability based on Egan’s rules, considering the metrics TPSA (polar surface area) and logP [50] in the computations (Table S3), and these were tagged as accepted, concerning the predicted descriptors. Nevertheless, due to these molecules’ many degrees of freedom, the number of rotatable bonds was higher than ten, augmenting the chance of binding on plasma proteins.

**Figure 6:**
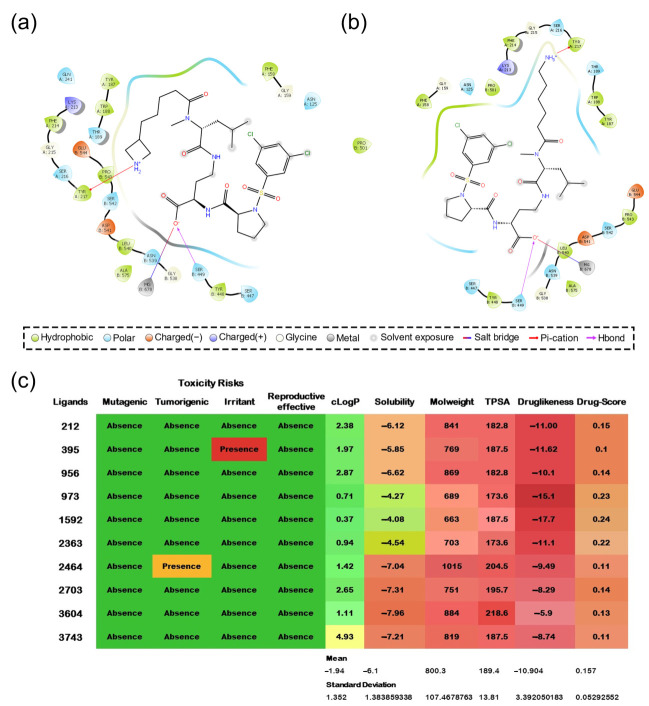
(**a**,**b**) Diagram of interactions of the complexes α4β1/Ligand 973 and α4β1/Ligand 1592, respectively. The prints were produced using the Maestro 11.8 software, with a 4 Å cut-off radius. ClogP: octanol-water partition coefficient; TPSA: topological surface area. (**c**) ADMET parameters analyzed in Osiris Property Explorer. The color range indicates that the stronger the green represents more desirable characteristics and the stronger the red represents characteristics more unsuitable for a drug.

We also calculated toxicity risks (mutagenic, tumorigenic, irritant) and physicochemical properties (ClogP, solubility, drug-likeness, and drug score) using the Osiris server (Figure 6c). Only two ligands showed potential toxicity risks, namely 395 (irritant) and 2464 (tumorigenic) compounds.

Molecules 973, 1592, and 2363 were the ones that exhibited the best solubility values (Figure 6c) without risk of toxicity. The molecule solubility is related to the diffusion of drugs from the administration site to the blood, given the drug interaction with plasma proteins [49]. Thus, the drug score is among the most appropriate for these three compounds.

Once all these analyses were carried out, we observed that ligands 973 and 1592 obtained better results from the ADMET analysis, as ascertained by all the programs applied (see the Supplementary Material).

Regarding the conserved interactions in VLA-4 (Figure 6a,b), 973 and 1592 formed a salt bridge between the carboxylate moiety and the MIDAS Mg^2+^ ion and a hydrogen bond with the hydroxyl group of Ser 449, belonging to the β chain of the integrin. The primary VLA-4 recognition core with the lead chemical BIO5192 includes these interactions. Furthermore, a new chemical fragment proposed by the de novo design caused a cation interaction between the NH_2_^+^ (973) and NH_3_^+^ (1592) groups and the Tyr 217 benzene ring, which belongs to the integrin α chain. Cation-π interactions prove necessary, especially with charged ligands [51,52]. Moreover, this receptor-ligand recognition helps modify or design new potential drug compounds [53].

These results show that we achieved our goal of reinforcing new contacts from the added fragments. Furthermore, we found interactions of these chemical scaffolds with hydrophobic amino acids of the α chain of VLA-4, constituting a crucial recognition center in the integrin pocket.

### 3.5. Stability and Dynamics of VLA-4 Ligands over Time

We applied the MD tool to study the stability of the integrin–ligand complexes and whether their interactions were maintained over time. We chose the lead compound BIO5192 and two of the most promising ligands, 1592 and 973, according to the results obtained, for the simulations.

**Figure 7:**
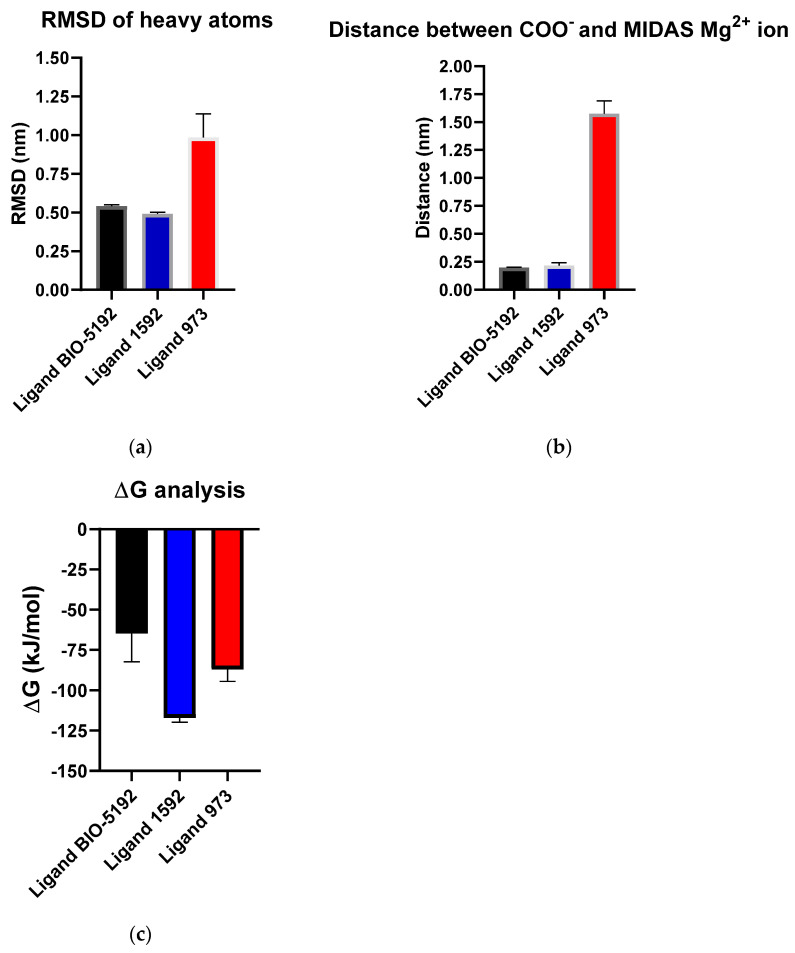
(**a**) Least-squares fit interpolation of heavy atom RMSD data for three replicas of 500 ns each of ligands BIO5192 (black) (mean = 0.5416 nm and SD = 0.1364 nm), 1592 (blue) (mean = 0.4916 nm and SD = 0.1819 nm), and 973 (red) (mean = 0.9845 nm and SD = 0.3165 nm). (**b**) Least-squares fit interpolation of distance data between the carboxylate oxygen and the VLA-4 integrin for three replicas of 500 ns each of ligands BIO5192 (black) (mean = 0.1986 nm and SD = 0.0062 nm), 1592 (blue) (mean = 0.2177 nm and SD = 0.1002 nm), and 973 (red) (mean = 1.5739 nm and SD = 0.4118 nm). (**c**) Least-squares fit interpolation of ΔG values for three replicas along the last 250 ns of each one for ligand BIO5192 (black) (mean = −64.85 kJ/mol and SD = 67.37 kJ/mol), 1592 (blue) (mean = −117.02 kJ/mol and SD = 31.33 kJ/mol), 973 (red) (mean = −86.94 kJ/mol and SD = 26.86 kJ/mol). Error bars indicate the standard deviation (SD). For all experiments, *p* > 0.99. The plots were generated in the GraphPad Prism version 8.0.2 for Windows, GraphPad Software, San Diego, California USA, www.graphpad.com.

Regarding the RMSD of the heavy atoms of the three ligands analyzed (Figure 7a), we noticed that, for the three replicates of 500 ns each, the average of the RMSD remained close to 0.5 nm for BIO5192 and for molecule 1592, indicating stability. However, ligand 973 presented a significant displacement of the interaction site in the integrin throughout the simulations (Figure S8a).

By monitoring the interaction between the MIDAS Mg^2+^ ion in VLA-4 and the carboxylate of each ligand (Figure 7b), we can observe the maintenance of this critical recognition over time for BIO5192 and 1592, with a mean distance of roughly 0.25 nm, reinforcing the relevance of the salt bridge in molecular recognition involving β1 integrins. Nevertheless, for the ligand 973, the interaction was lost, probably because the initial docking pose was not the most appropriate. Therefore, the RMSD values remained high throughout the simulations (see the Supplementary Material).

The binding free energy results show that ligand 1592 had a lower mean value of ΔG than the other compounds, suggesting that it is the molecule with the highest affinity with VLA-4. The lower ΔG standard deviation values observed for ligands 1592 and 973, when compared with BIO5192, indicate greater stability of these molecules at the VLA-4 interaction site along the calculated trajectory (last 250 ns of the simulations).

## 4. Conclusions

This study presents the discovery of prototypic compounds that could be used as a basis for the development of new drugs for the treatment of chronic inflammatory diseases, namely, multiple sclerosis, rheumatoid arthritis, and IBDs, among others.

Using comparative modeling, molecular docking, and fragment-based de novo design, we determined that the new BIO5192 derivatives have a greater estimated affinity for the α4β1 integrin than the lead drug, suggesting a possible inhibitory capability for these ligands.

Furthermore, the ADMET and the interactions with the target analyses indicated the molecules 973 and 1592 as promising VLA-4 inhibitors. Such compounds presented low toxicity risks and increased the number of interactions with VLA-4, mainly in the region where the new fragments were added.

Finally, at the end of the molecular dynamics simulations, we found that the ligand 1592 showed stable behavior at the VLA-4 interaction site, indicating similarity with the lead compound BIO5192. Furthermore, from the binding free energy calculations, 1592 showed a higher affinity to VLA-4 than the other molecules studied. Overall, our data strongly indicate that ligand 1592 represents a possible candidate for a VLA-4 inhibitory drug.

In this way, through the application of several computational techniques, we were able to filter plenty of molecules so that, in the end, we could find a strong drug candidate. Accordingly, our results shed light on new perspectives in studying different binding modes of integrin small-molecule inhibitors and allosteric sites in the protein receptor, which still lacks more details in the scientific community.

## Figures and Tables

**Table 1:** Calculated affinity data using the RACHEL module. Scores are shown in pKi units.

Derivatization I		Derivatization II		Derivatization III	
Ligand	Score	Ligand	Score	Ligand	Score
**171**	8.31	212	**9.08**	2048	8.48
**72**	8.22	395	**8.99**	1229	8.45
**177**	7.83	2363	**8.93**	1634	8.38
**172**	7.82	2703	**8.87**	1188	8.32
**75**	7.49	3743	**8.73**	955	8.20
**83**	7.47	1592	**8.63**	985	8.03
**160**	7.46	973	**8.60**	1206	7.98
**245**	7.46	956	**8.58**	2223	7.79
**45**	7.45	2464	**8.51**	1	7.74
**259**	7.45	3604	**8.45**	1114	7.73

Note: The column highlighted in bold represents the derivatization that resulted in molecules with higher scores.

## Data Availability

The datasets analyzed during this current study are available from the corresponding author on reasonable request.

## Supplementary Materials

The following supporting information can be downloaded at: https://www.mdpi.com/article/10.3390/life12070932/s1, Figure S1: Alignment of the templates (upper sequences) and the models (lower sequences) for (a) α4, (b) αV, and (c) β1, Figure S2: Ramachandran plots of the best (a) α4β1 and (b) αVβ1 models. Images produced using the server Procheck, Figure S3: Analyses performed using Verify3D server on the compatibility of the three-dimensional models of (a) and (b) α4β1; (c) and (d) αVβ1. The yellow line represents the minimum necessary value, measured for each residue, for a structure to be considered compliant, Figure S4: Z-score graphs from the ProSA-web server designating the quality of the models generated for (a) and (b) α4β1; (c) and (d) αVβ1. The black dot represents where the models were located among the experimentally determined protein structures, Figure S5: All nine binding modes of BIO1211 at the αVβ1 pocket. The red sphere represents the Mg2+ MIDAS ion. The integrin chains are depicted as alpha (green) and beta (cyan). Image produced using PyMOL, Figure S6: All nine binding modes of BIO5192 at the (a) α5β1 and (b) αVβ1 pocket. The red sphere represents the Mg2+ MIDAS ion. The integrin chains are represented by the colors yellow, cyan, and green, as shown in the respective figures. Image produced using PyMOL, Figure S7: Protonated chemical structures of the ten best ligands designed, with the designation of their respective codes. were obtained by using the Maestro 11.8 software, Figure S8: (a) Data of RMSD of heavy atoms of ligand 973, over 500 ns of simulation, according to replicas indicated in black (Replica 1), blue (Replica 2), and red (Replica 3). (b) Distances between the carboxylate oxygen of ligand 973 and the MIDAS Mg2+ ion of VLA-4, over 500 ns of simulation, according to the replicas indicated in black (Replica 1), blue (Replica 2), and red (Replica 3). The plots were generated in the GraphPad Prism 8.0.2 software, Table S1: Evaluation of the ten best-ranked VLA-4 ligands according to the Vina score and the activities measured by the corresponding IC50 values (Half Maximal Inhibitory Concentration). The activity information was taken from the Chembl database, Table S2: Evaluation of the Lipinski’s Rule of 5 based on the ClogP/CMR model, Table S3: ADMET parameters analyzed by FAF-Drugs3.

## List of Abbreviations

ADMET	Absorption, distribution, metabolism, elimination, and toxicity
AUC	Area under the curve
ECM	Extracellular matrix
EF	Enrichment factor
FBDD	Fragment-based drug discovery
FDA	Food and Drug Administration
MD	Molecular dynamics
MIDAS	Metal ion-dependent adhesion site
NCBI	National Center for Biotechnology Information
PDB	Protein Data Bank
PME	Particle mesh Ewald
PUPA	[N′-(2-methyl phenyl) ureido] phenyl acetyl
RACHEL	Real-time Automated Combinatorial Heuristic Enhancement of Lead compounds
ROC	Receiver operating characteristics
SD	Standard deviation
VLA-4	Very late antigen-4
ZBB	ZINC building blocks
