# Supplementary material for: Development of New Potential Inhibitors of β1 Integrins through In Silico Methods—Screening and Computational Validation
Source: Life (Basel). 2022 Jun 22;12(7):932. doi: 10.3390/life12070932 (PMC9325263; doi:10.3390/life12070932)
Supplement: Supplementary file 1 [file life-12-00932-s001.zip › life-1739917-supplementary.pdf]

Development of new potential inhibitors of β1 integrins through in silico methods – Screening and computational validation

Supplementary Material

α4

```
---------------------------------YNVDTESALLYQGPHNTLFGYSVVLHS
HGANRWLLVGAPTANWLANASVINPGAIYRCRIGKNPGQTCEQLQLGSPNGEPCGKTCLE
ERDNQWLGVTLSRQPGENGSIVTCGHRWKNIFYIKNENKLPTGGCYGVPPDLRTELSKRI
APCYQDYVKKFGENFASCQAGISSFYTKDLIVMGAPGSSYWTGSLFVYNITTNKYKAFLD
KQNQVKFGSYLGYSVGAGHFRSQHTTEVVGGAPQHEQIGKAYIFSIDEKELNILHEMKGK
KLGSYFGASVCAVDLNADGFSDLLVGAPMQSTIREEGRVFVYINSGSGAVMNAMETNLVG
SDKYAARFGESIVNLGDIDNDGFEDVAIGAPQEDDLQGAIYIYNGRADGISSTFSQRIEG
LQISKSLSMFGQSISGQIDADNNGYVDVAVGAFRSDSAVLLRTRPV...

---------------------------------YNVDTESALLYQGPHNTLFGYSVVLHS
HGANRWLLVGAPTANWLANASVINPGAIYRCRIGKNPGQTCEQLQLGSPNGEPCGKTCLE
ERDNQWLGVTLSRQPGENGSIVTCGHRWKNIFYIKNENKLPTGGCYGVPPDLRTELSKRI
APCYQDYVKKFGENFASCQAGISSFYTKDLIVMGAPGSSYWTGSLFVYNITTNKYKAFLD
KQNQVKFGSYLGYSVGAGHFRSQHTTEVVGGAPQHEQIGKAYIFSIDEKELNILHEMKGK
KLGSYFGASVCAVDLNADGFSDLLVGAPMQSTIREEGRVFVYINSGSGAVMNAMETNLVG
SDKYAARFGESIVNLGDIDNDGFEDVAIGAPQEDDLQGAIYIYNGRADGISSTFSQRIEG
LQISKSLSMFGQSISGQIDADNNGYVDVAVGAFRSDSAVLLRTRPV...
```

(a)

αV

```
FNLDVDSPAEYSGPEGSYFGFAVDFFVPSASSRMFLLVGAPKANTTQPGIVEGGQVLKCDWSSTRRCQPI
EFDATGNRDYAKDDPLEFKSHQWFGASVRSKQDKILACAPLYHWRTEMKQEREPVGTCFLQDGTKTVEYA
PCRSQDIDADGQGFCQGGFSIDFTKADRVLLGGPGSFYWQGQLISDQVAEIVSKYDPNVYSIKYNNQLAT
RTAQAIFDDSYLGYSVAVGDFNGDGIDDFVSGVPRAARTLGMVYIYDGKNMSSLYNFTGEQMAAYFGFSV
AATDINGDDYADVFIGAPLFMDRGSDGKLQEVGQVSVSLQRASGDFQTTKLNGFEVFARFGSAIAPLGDL
DQDGFNDIAIAAPYGGEDKKGIVYIFNGRSTGLNAVPSQILEGQWAARSMPPSFGYSMKGATDIDKNGYP
DLIVGAFGVDRAILYRARPVI....

FNLDVDSPAEYSGPEGSYFGFAVDFFVPSASSRMFLLVGAPKANTTQPGIVEGGQVLKCDWSSTRRCQPI
EFDATGNRDYAKDDPLEFKSHQWFGASVRSKQDKILACAPLYHWRTEMKQEREPVGTCFLQDGTKTVEYA
PCRSQDIDADGQGFCQGGFSIDFTKADRVLLGGPGSFYWQGQLISDQVAEIVSKYDPNVYSIKYNNQLAT
RTAQAIFDDSYLGYSVAVGDFNGDGIDDFVSGVPRAARTLGMVYIYDGKNMSSLYNFTGEQMAAYFGFSV
AATDINGDDYADVFIGAPLFMDRGSDGKLQEVGQVSVSLQRASGDFQTTKLNGFEVFARFGSAIAPLGDL
DQDGFNDIAIAAPYGGEDKKGIVYIFNGRSTGLNAVPSQILEGQWAARSMPPSFGYSMKGATDIDKNGYP
DLIVGAFGVDRAILYRARPVI....
```

(b)

β1

```
---------------------PIDLYYLMDLSYSMKDDLENVKSLGTDLMNEMRRITSDF
RIGFGSFVEKTVMPYISTTPAKLRNPCTSEQNCTTPFSYKNVLSLTNKGEVFNELVGKQR
ISGNLDSPEGGFDAIMQVAVCGSLIGWRNVTRLLVFSTDAGFHFAGDGKLGGIVLPNDGQ
CHLENNMYTMSHYYDYPSIAHLVQKLSENNIQTIFAVTEEFQPVYKELKNLIPKSAVGTL
SANSSNVIQLIIDAYNSLSSE..*

---------------------PIDLYYLMDLSYSMKDDLENVKSLGTDLMNEMRRITSDF
RIGFGSFVEKTVMPYISTTPAKLRNPCTSEQNCTSPFSYKNVLSLTNKGEVFNELVGKQR
ISGNLDSPEGGFDAIMQVAVCGSLIGWRNVTRLLVFSTDAGFHFAGDGKLGGIVLPNDGQ
CHLENNMYTMSHYYDYPSIAHLVQKLSENNIQTIFAVTEEFQPVYKELKNLIPKSAVGTL
SANSSNVIQLIIDAYNSLSSE..*
```

(c)

Figure S1. Alignment of the templates (upper sequences) and the models (lower

sequences) for (a) α4, (b) αV, and (c) β1.

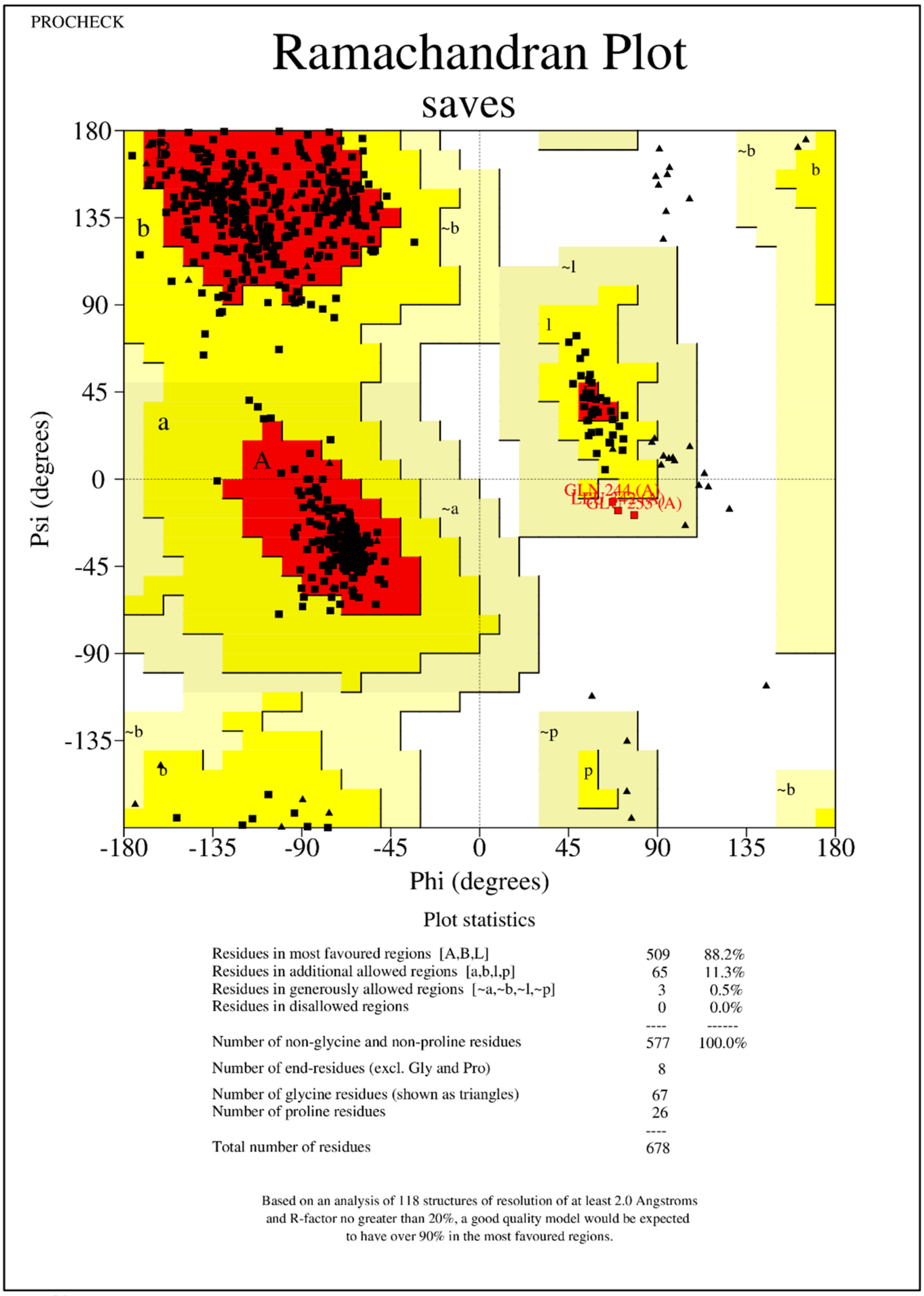

saves_01.ps

(a)

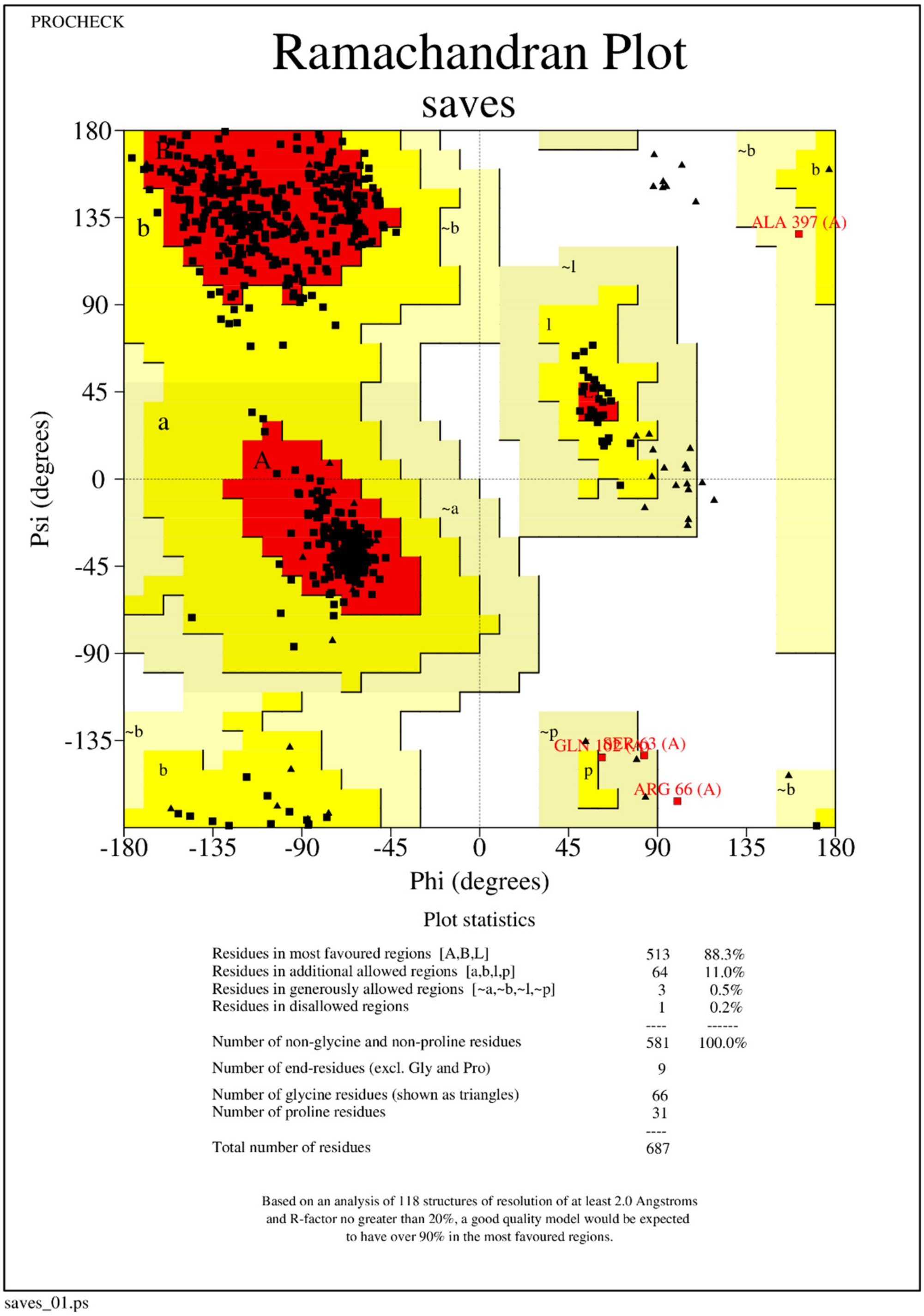

(b)

Figure S2. – Ramachandran plots of the best (a) α4β1 and (b) αVβ1 models. Images produced using the server Procheck.

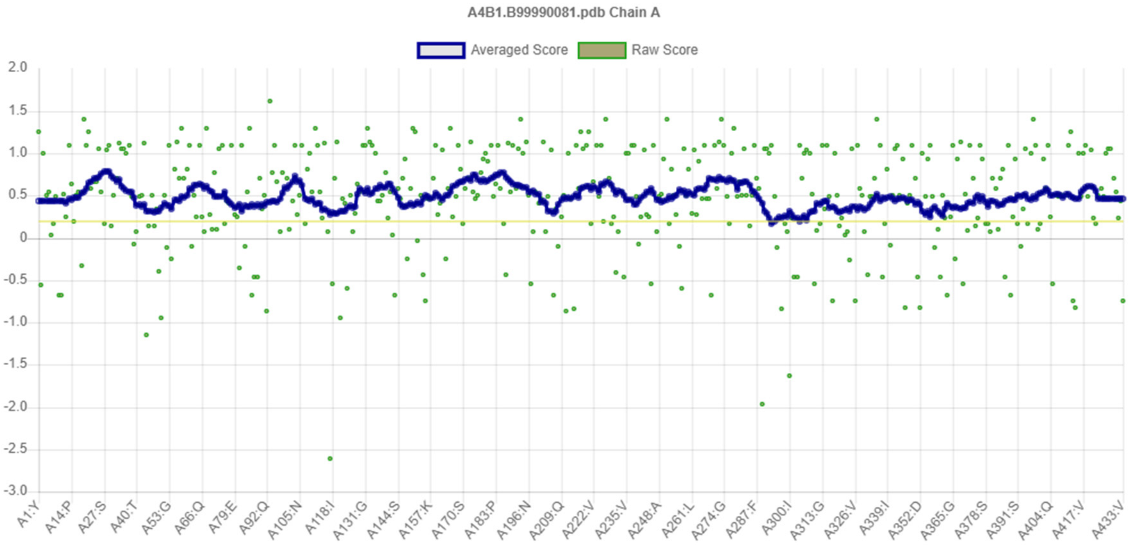
A4B1.B99990081.pdb Chain A
Averaged Score
Raw Score
2.0
1.5
1.0
0.5
0
-0.5
-1.0
-1.5
-2.0
-2.5
-3.0
A1:Y A14:P A27:S A40:T A53:G A66:Q A79:E A92:Q A105:N A118:I A131:G A144:S A157:K A170:S A183:P A196:N A209:Q A222:V A235:V A248:A A261:L A274:G A287:F A300:I A313:G A326:V A339:I A352:D A365:G A378:S A391:S A404:Q A417:V A433:V

(a)

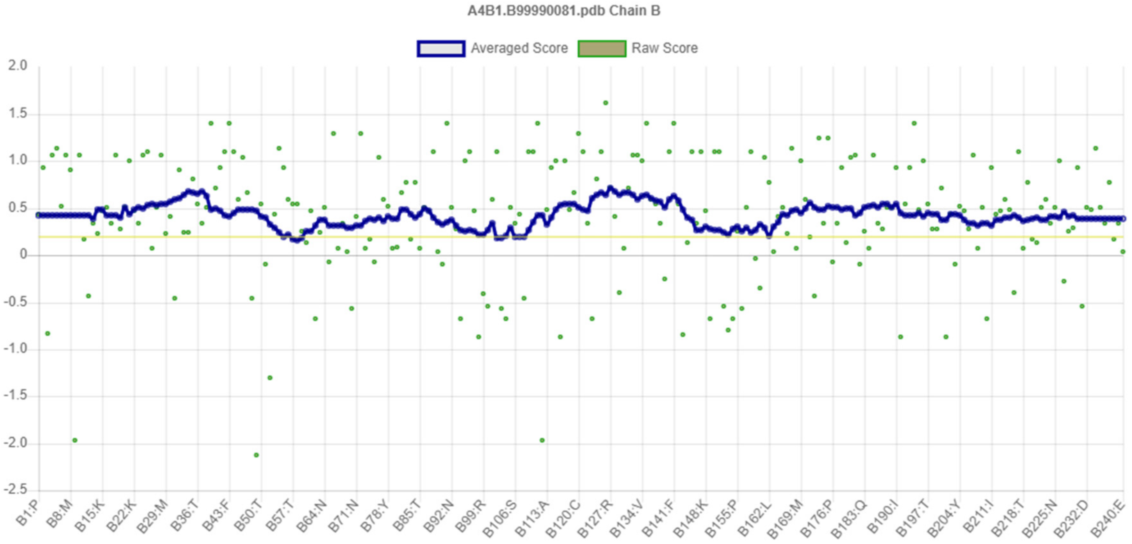
A4B1.B99990081.pdb Chain B
Averaged Score
Raw Score
2.0
1.5
1.0
0.5
0
-0.5
-1.0
-1.5
-2.0
-2.5
B1:P B8:M B15:K B22:K B29:M B36:T B43:F B50:T B57:T B64:N B71:N B78:Y B85:T B92:N B99:R B106:S B113:A B120:C B127:R B134:V B141:F B148:K B155:P B162:L B169:M B176:P B183:Q B190:I B197:T B204:Y B211:I B218:T B225:N B232:D B240:E

(b)

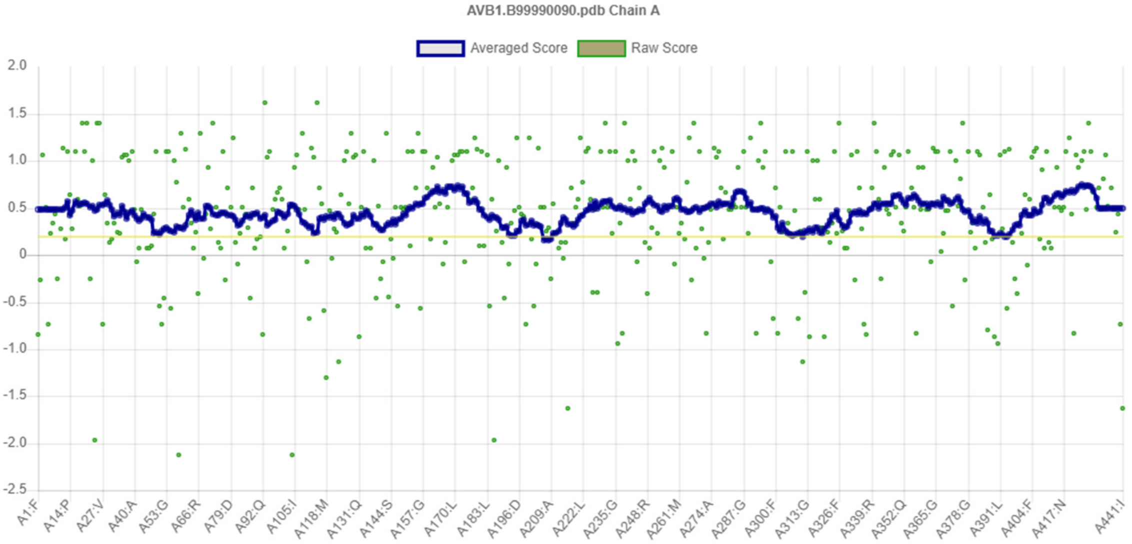
AVB1.B99990090.pdb Chain A
Averaged Score
Raw Score
2.0
1.5
1.0
0.5
0
-0.5
-1.0
-1.5
-2.0
-2.5
A1:F A14:P A27:V A40:A A53:G A66:R A79:D A92:Q A105:I A118:M A131:Q A144:S A157:G A170:L A183:L A196:D A209:A A222:L A235:G A248:R A261:M A274:A A287:G A300:F A313:G A326:F A339:R A352:Q A365:G A378:G A391:L A404:F A417:N A441:I

(c)

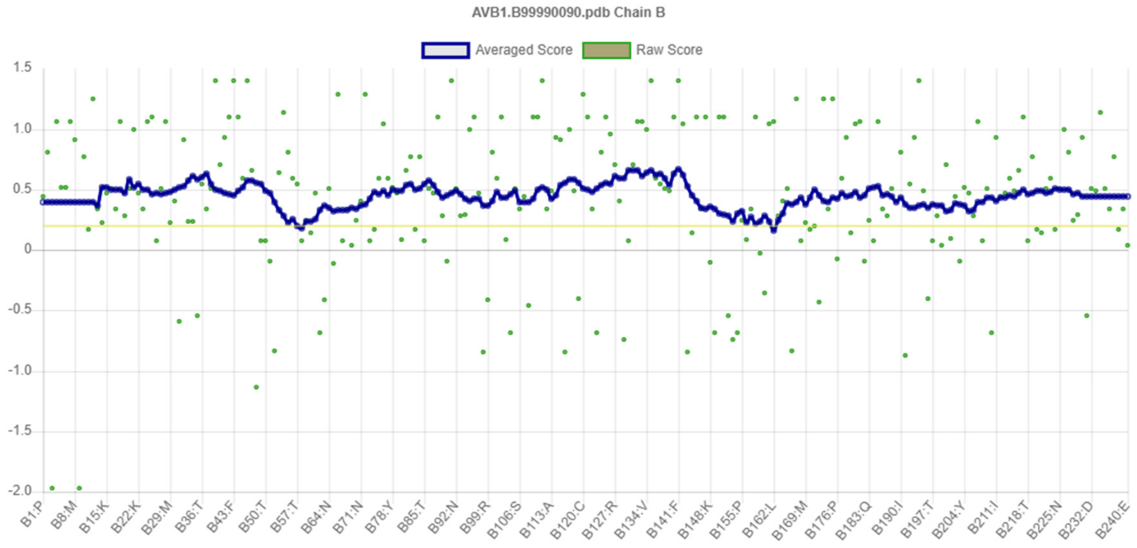

(d)

Figure S3. – Analyses performed using Verify3D server on the compatibility of the three-dimensional models of (a) and (b) α4β1; (c) and (d) αVβ1. The yellow line represents the minimum necessary value, measured for each residue, for a structure to be considered compliant.

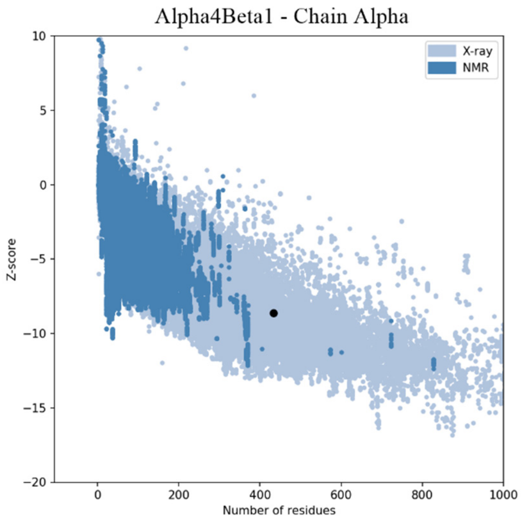

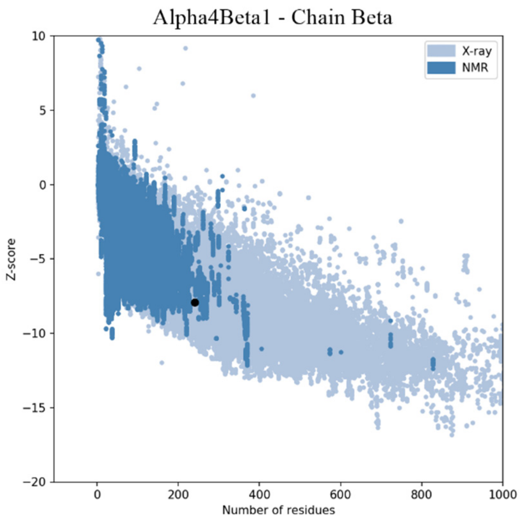

(a) (b)

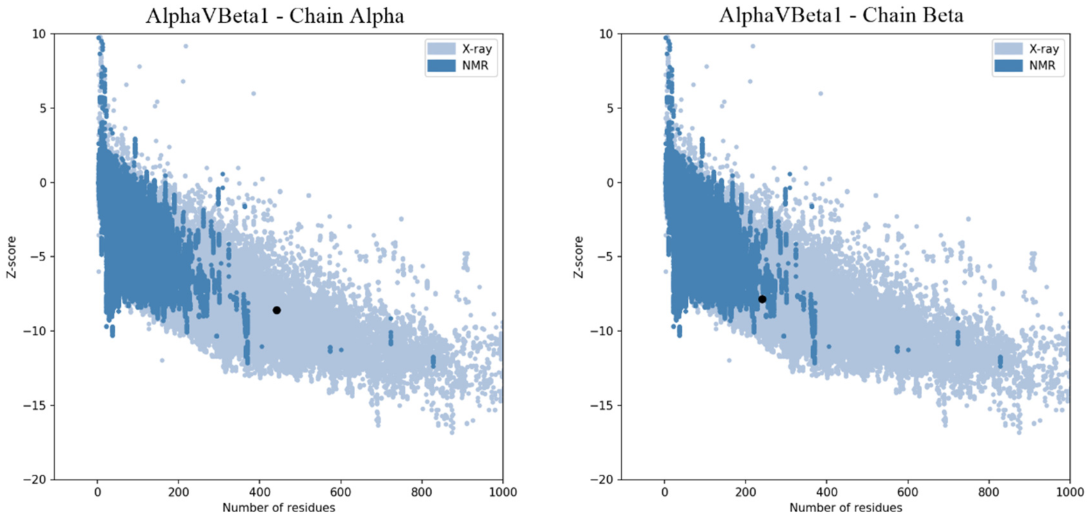

(c) (d)

Figure S4. – Z-score graphs from the ProSA-web server designating the quality of the models generated for (a) and (b) α4β1; (c) and (d) αVβ1. The black dot represents where the models were located among the experimentally determined protein structures.

Table S1. Evaluation of the ten best-ranked VLA-4 ligands according to the Vina score and the activities measured by the corresponding IC50 values (Half Maximal Inhibitory Concentration). The activity information was taken from the Chembl database.

**Ranking**	**Vina Score (kcal/mol)**	**Chembl Reference**	**IC50 (nM)**	**Activity**
1	-10.2	1940754	4.7	Active
2	-10.2	285374	11	Active
3	-9.9	2391499	430	Decoy
4	-9.9	1940758	43	Active
5	-9.9	2391310	760	Decoy
6	-9.9	280746	3	Active
7	-9.9	567180	8.5	Active
8	-9.8	572802	32	Active
9	-9.8	569302	0.2	Active
10	-9.8	566091	2.7	Active

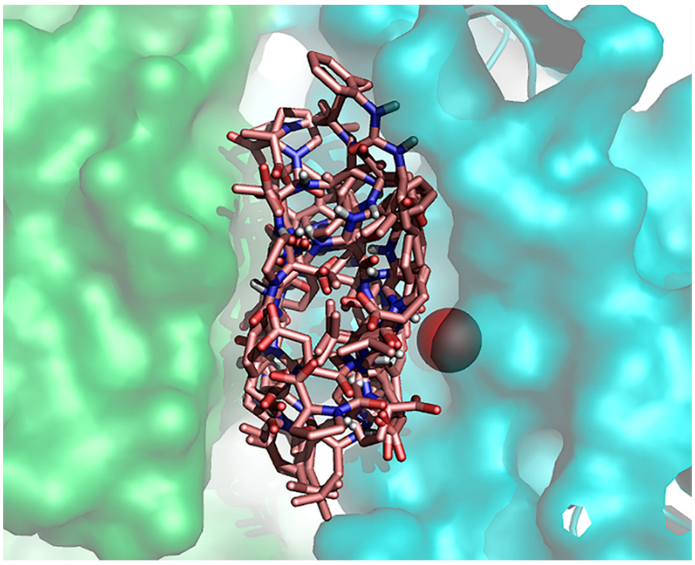

Figure S5. – All nine binding modes of BIO1211 at the αVβ1 pocket. The red sphere represents the Mg^{2+} MIDAS ion. The integrin chains are depicted as alpha (green) and beta (cyan). Image produced using PyMOL.

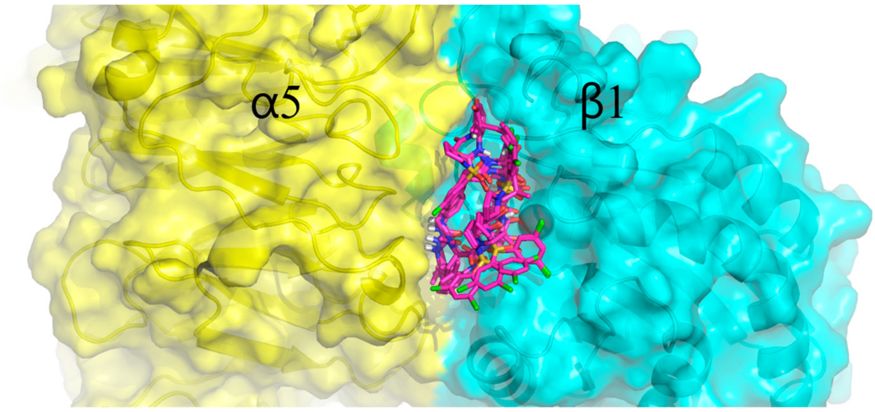

(a)

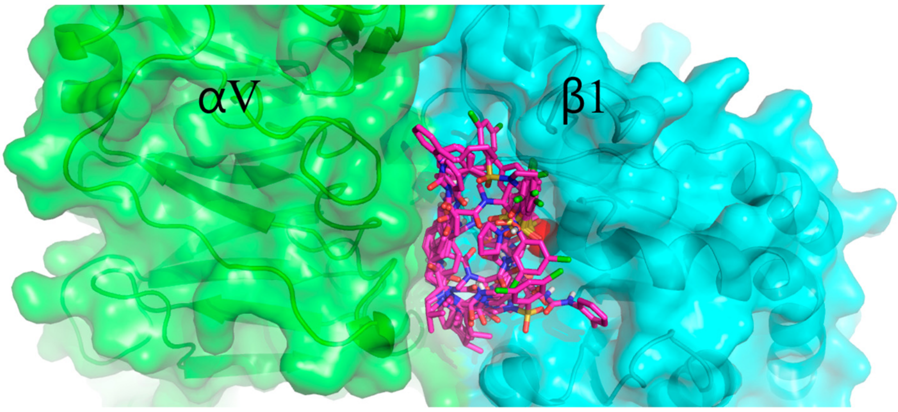

(b)

Figure S6. – All nine binding modes of BIO5192 at the (a) α5β1 and (b) αVβ1 pocket. The red sphere represents the Mg^{2+} MIDAS ion. The integrin chains are represented by the colors yellow, cyan, and green, as shown in the respective figures. Image produced using PyMOL.

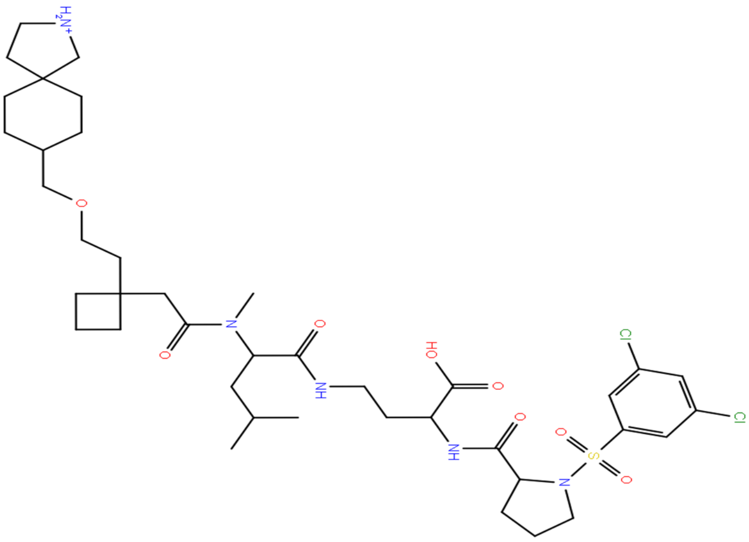

212

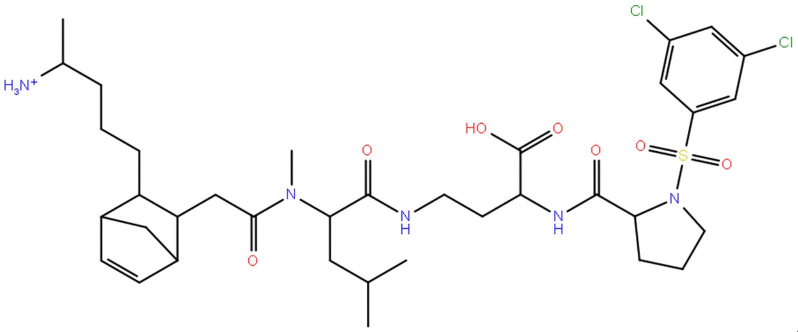

395

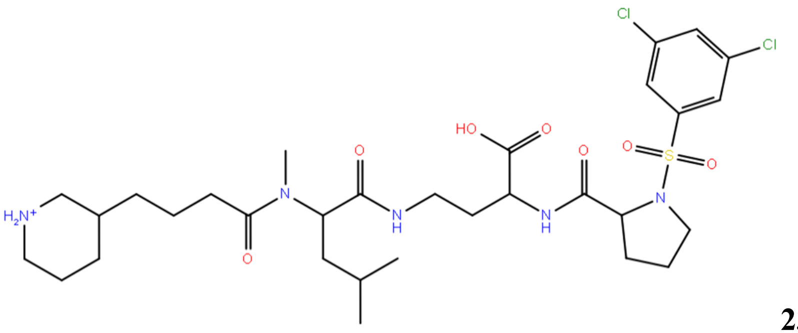

2363

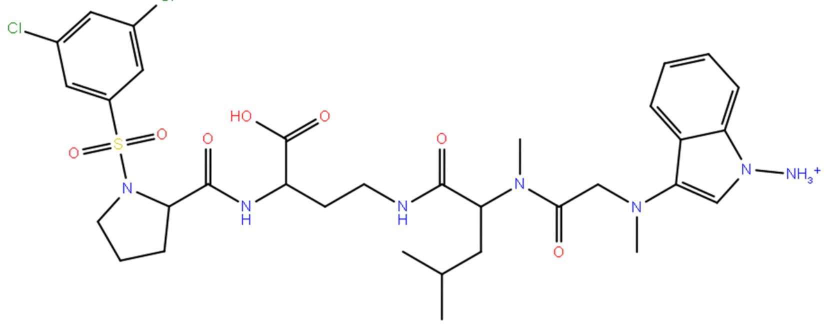

2703

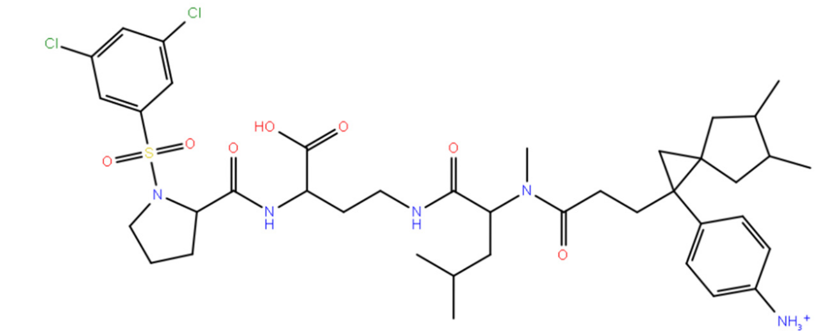

3743

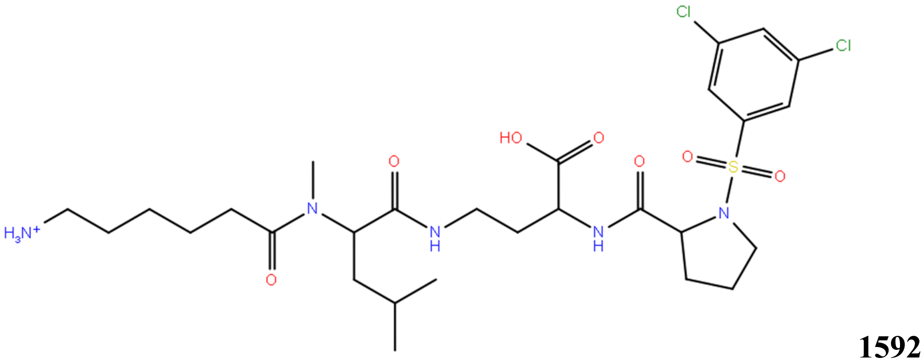

1592

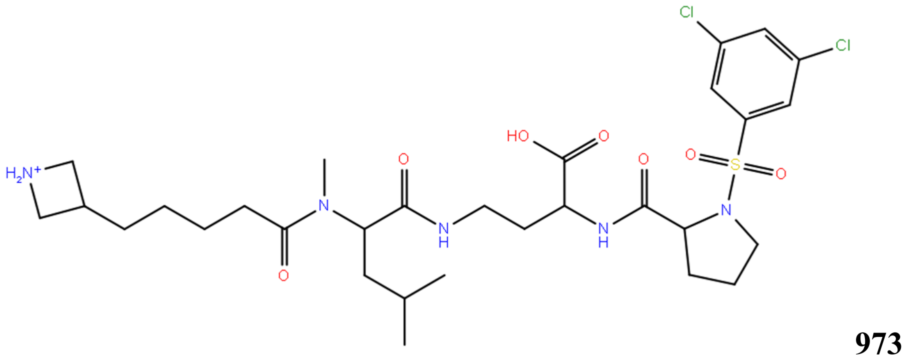

973

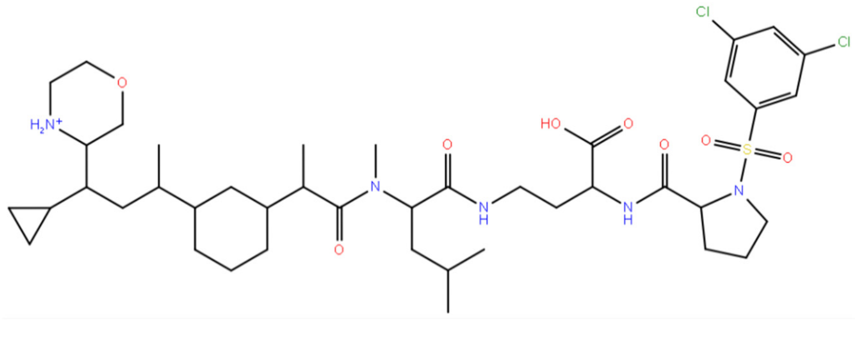

956

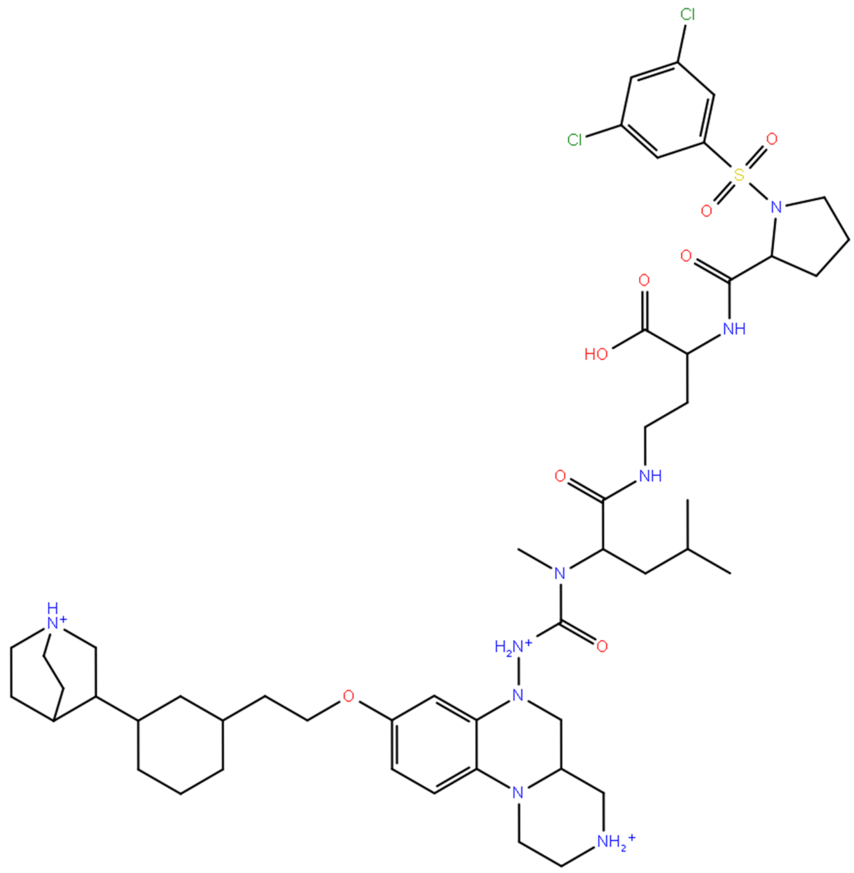

2464

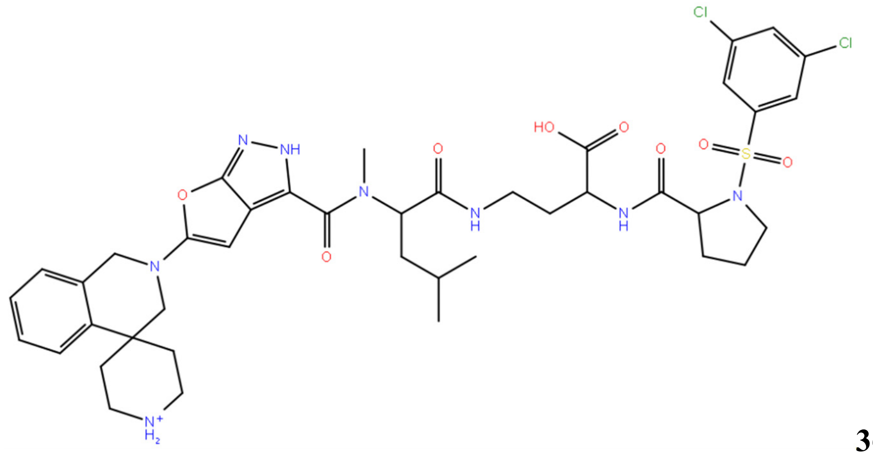

3604

Figure S7. – Protonated chemical structures of the ten best ligands designed, with the designation of their respective codes. Figures were obtained by using the Maestro 11.8 software.

Table S2. Evaluation of the Lipinski's Rule of 5 based on the ClogP/CMR model

Ligands	Lipinski's Parameters			
	HBA	HBD	MW	CLOGP
**212**	13	4	842.91	3.43
**395**	12	4	770.80	3.56
**956**	13	4	870.96	5.26
**973**	12	4	690.67	1.29
**1592**	12	4	664.64	0.82
**2363**	12	4	704,.70	1.88
**2464**	17	5	1017.11	5.70
**2703**	14	4	752.70	5.60
**3604**	16	5	885.85	2.50
**3743**	12	4	820.86	7.02

HBA: Hydrogen bond acceptor; HBD: hydrogen bond donor; MW: Molecular Weight; ClogP: Octanol-water partition coefficient based on the ClogP/CMR model.

Table S3. ADMET parameters analyzed by FAF-Drugs3

Ligands	TPSA (Å²)	Rotatable Bonds	log P	Oral Bioavailability EGAN
**212**	190	19	3.40	Good
**395**	192	18	2.72	Good
**956**	190	19	5.21	Good
**973**	181	17	1.26	Good
**1592**	192	17	0.53	Good
**2363**	181	16	1.43	Good
**2464**	213	18	5.21	Good
**2703**	199	15	4.43	Good
**3604**	226	14	3.33	Good
**3743**	190	16	6.99	Good

TPSA: Topological Surface Area; log P: Octanol-water partition coefficient. Note: Egan rule considers, in the calculations, the parameters TPSA (Polar Surface Area) and log P.

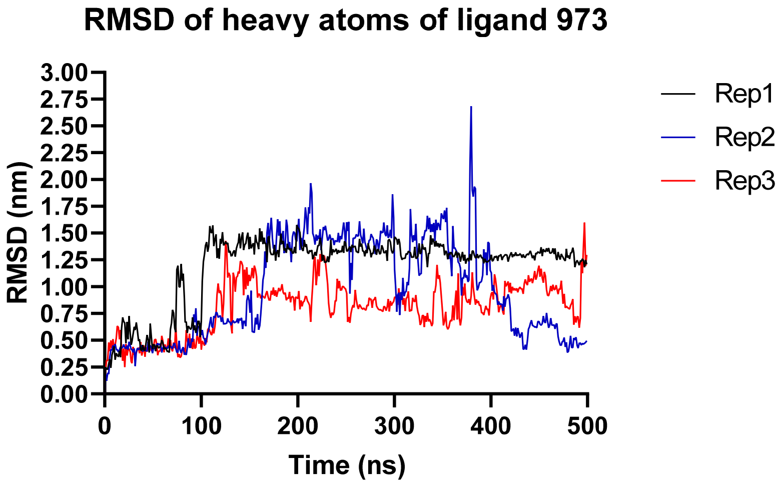

(a)

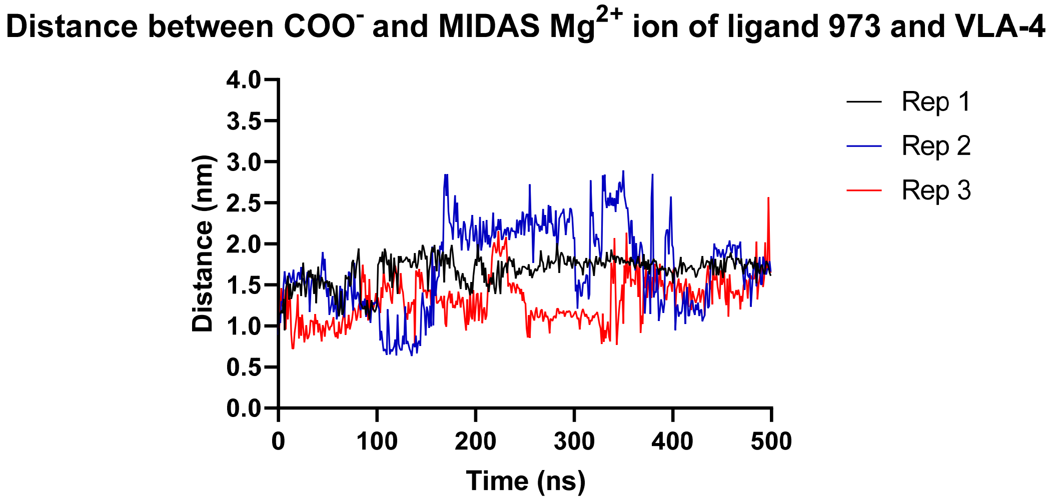

(b)

Figure S8. – (a) Data of RMSD of heavy atoms of ligand 973, over 500 ns of simulation, according to replicas indicated in black (Replica 1), blue (Replica 2), and red (Replica 3). (b) Distances between the carboxylate oxygen of ligand 973 and the MIDAS Mg^{2+} ion of VLA-4, over 500 ns of simulation, according to the replicas indicated in black (Replica 1), blue (Replica 2), and red (Replica 3). The plots were generated in the GraphPad Prism 8.0.2 software.
